# Prognostic role of short-term heart rate variability and deceleration/acceleration capacities of heart rate in extensive-stage small cell lung cancer

**DOI:** 10.3389/fphys.2023.1277383

**Published:** 2023-11-08

**Authors:** Shuang Wu, Weizheng Guan, Huan Zhao, Guangqiao Li, Yufu Zhou, Bo Shi, Xiaochun Zhang

**Affiliations:** ^1^ School of Medicine, Yangzhou University, Yangzhou, Jiangsu, China; ^2^ Department of Radiation Oncology, First Affiliated Hospital, Bengbu Medical College, Bengbu, Anhui, China; ^3^ School of Medical Imaging, Bengbu Medical College, Bengbu, Anhui, China; ^4^ Anhui Key Laboratory of Computational Medicine and Intelligent Health, Bengbu Medical College, Bengbu, Anhui, China; ^5^ Department of Oncology, Yangzhou Hospital of Traditional Chinese Medicine, Yangzhou, Jiangsu, China

**Keywords:** extensive-stage small cell lung cancer, autonomic modulation, heart rate variability, deceleration/acceleration capacities of heart rate, prognosis

## Abstract

**Background:** Prior research suggests that autonomic modulation investigated by heart rate variability (HRV) might act as a novel predictive biomarker for cancer prognosis, such as in breast cancer and pancreatic cancer. It is not clear whether there is a correlation between autonomic modulation and prognosis in patients with extensive-stage small cell lung cancer (ES-SCLC). Therefore, the purpose of the study was to examine the association between short-term HRV, deceleration capacity (DC) and acceleration capacity (AC) of heart rate and overall survival in patients with ES-SCLC.

**Methods:** We recruited 40 patients with ES-SCLC, and 39 were included in the final analysis. A 5-min resting electrocardiogram of patients with ES-SCLC was collected using a microelectrocardiogram recorder to analyse short-term HRV, DC and AC. The following HRV parameters were used: standard deviation of the normal-normal intervals (SDNN) and root mean square of successive interval differences (RMSSD). Overall survival of patients with ES-SCLC was defined as time from the date of electrocardiogram measurement to the date of death or the last follow-up. Follow-up was last performed on 07 June 2023. There was a median follow-up time of 42.2 months.

**Results:** Univariate analysis revealed that the HRV parameter SDNN, as well as DC significantly predicted the overall survival of ES-SCLC patients (all *p* < 0.05). Multivariate analysis showed that the HRV parameters SDNN (hazard ratio = 5.254, 95% CI: 1.817–15.189, *p* = 0.002), RMSSD (hazard ratio = 3.024, 95% CI: 1.093–8.372, *p* = 0.033), as well as DC (hazard ratio = 3.909, 95% CI: 1.353–11.293, *p* = 0.012) were independent prognostic factors in ES-SCLC patients.

**Conclusion:** Decreased HRV parameters (SDNN, RMSSD) and DC are independently associated with shorter overall survival in ES-SCLC patients. Autonomic nervous system function (assessed based on HRV and DC) may be a new biomarker for evaluating the prognosis of patients with ES-SCLC.

## Introduction

Lung cancer is the leading cause of cancer-related mortality worldwide (Sung et al., 2021). Small cell lung cancer (SCLC) accounts for approximately 15% of all lung cancer cases (Wang et al., 2020). SCLC is characterized by early invasion and rapid metastasis and is the most aggressive histologic subtype of lung cancer; most SCLC patients are diagnosed with extensive-stage SCLC (ES-SCLC) (Mak et al., 2019; Wang et al., 2019). Although SCLC is highly sensitive to chemotherapy and radiotherapy, the prognosis of SCLC patients remains poor, with a 5-year survival rate of less than 10% (Foster et al., 2009; Janssen-Heijnen et al., 2012; Dayen et al., 2017). In addition to standardized treatment for SCLC patients, clinicians also need more prognostic parameters to provide a theoretical basis for individualized treatment and risk stratification.

The autonomic nervous system (ANS), divided into the sympathetic nervous system (SNS) and the parasympathetic nervous system (PNS), has been correlated with the occurrence and development of malignant tumours (Simó et al., 2018; Faulkner et al., 2019). Chronic or acute stress activates the SNS, which releases catecholamines into tissues and the circulation to affect host physiology in a wide range of ways (Mueller, 2022). Published studies have shown that the SNS sends signals to adrenergic receptors, which may induce tumour-promoting inflammation, inhibit antitumour immunity, and enhance the development of metastasis (Sloan et al., 2010; Huan et al., 2017; Qiao et al., 2018). High PNS activity may affect tumour progression by inhibiting tumour-related inflammatory responses, which may be related to the activation of the cholinergic anti-inflammatory pathway to reduce systemic inflammatory cytokines (Pavlov and Tracey, 2005; Williams et al., 2019; Zhang et al., 2021). In the clinical setting, several noninvasive and easily accessible indicators of ANS function, such as heart rate variability (HRV), deceleration capacity (DC) and acceleration capacity (AC) of heart rate, have been used, enabling assessment of the link between ANS function and mortality risk in patients with malignant tumours (Boehm et al., 2018; Kloter et al., 2018).

HRV is the variation in duration of successive R-R intervals, and it reflects ANS regulation (Camm et al., 1996; Lombardi and Stein, 2011). Most previous studies have shown that HRV has predictive value in the overall survival of cancer patients; that is, ANS dysfunction (characterized by decreased HRV parameters) may predict shorter survival of cancer patients (Ciurea et al., 2021; Wang et al., 2021; Wu et al., 2022). For example, the HRV parameter “standard deviation of the normal-normal intervals” (SDNN) is independently correlated with overall survival in patients with hepatocellular carcinoma (Ciurea et al., 2021). The HRV indicator “root mean square of successive interval differences” (RMSSD) is an independent prognostic factor in lung cancer patients with brain metastases (Wu et al., 2022).

DC and AC are new noninvasive techniques that can be used to evaluate autonomic modulation. DC and AC are measures of all deceleration-related and acceleration-related oscillations of time intervals between heartbeats (Bauer et al., 2006a; Zou et al., 2016). DC has been widely used in many studies related to heart disease, and these studies have confirmed that DC has great predictive value in mortality risk stratification in patients with myocardial infarction, heart failure or coronary artery disease (Arsenos et al., 2016; Wang et al., 2017; Rizas et al., 2018). Previous studies have also shown that AC can be used to assess ANS impairment in dilated cardiomyopathy patients and may be predictive of cardiac death (Zou et al., 2016; Yang et al., 2018). A recent study that examined the association between DC and survival in patients with malignant tumours found that DC assessed the risk stratification of emergency patients with malignant disease (Boehm et al., 2018). In addition, studies have shown that DC is an effective predictor of the risk of antineoplastic treatment-related cardiotoxicity in breast cancer patients (Feng and Yang, 2015; Feng et al., 2021). Although DC and AC may have higher specificity and sensitivity in patient stratification based on risk of death from heart disease than traditional detection techniques, they are rarely used in studies related to malignant tumours. Therefore, DC and AC deserve to be used in more clinical studies of malignant tumours.

Currently, the effect of short-term HRV, DC and AC on the prognosis of ES-SCLC patients remains unclear. Therefore, the aim of this study was to examine the prognostic role of short-term HRV, DC and AC in ES-SCLC patients.

## Methods

### Subjects

In this study, we enrolled patients diagnosed with ES-SCLC from October 2019 to April 2021 at the First Affiliated Hospital of Bengbu Medical College. The inclusion criteria were as follows: (1) histological or cytological confirmation of SCLC and (2) stage ES-SCLC. The exclusion criteria were as follows: (1) complications with other types of malignant tumours; (2) use of antiarrhythmic drugs or beta-blockers; (3) previous cardiac diseases (i.e., coronary heart disease, myocardial infarction, and atrial fibrillation); (4) chemotherapy, radiotherapy or surgery within 3 weeks before electrocardiogram data collection; and (5) absence of clinical data or overall survival. The Medical Ethics Committee of the local hospital approved the study (registration number: 2019KY031), and all patients voluntarily signed informed consent.

### Data collection

A micro-electrocardiogram recorder (HeaLink-R211B; HeaLink Ltd., Bengbu, China) was used to collect a 5-min resting electrocardiogram for ES-SCLC patients in the supine position with a 400 Hz sampling rate and V6-lead. Clinical information and overall survival of ES-SCLC patients were collected from medical records or by phone call. A patient’s overall survival was determined from the date of electrocardiogram measurement to the date of death or last follow-up. The last follow-up date was on 07 June 2023.

### Heart rate variability and deceleration/acceleration capacities of heart rate analysis

In this study, electrocardiogram R-peaks were detected using the Pan-Tompkins algorithm (Pan and Tompkins, 1985). The electrocardiogram-derived respiratory method was used to calculate the estimated values for respiratory rate (Moody et al., 1985). Technical and physiological artefacts were automatically corrected by the threshold-based automatic artefact correction algorithm in Kubios software (Tarvainen et al., 2014). The following common HRV parameters were used: SDNN and RMSSD. DC and AC were calculated using the phase-rectified signal averaging technique. In the first step, to detect decelerating and accelerating anchors, the R-R interval time series were scanned for longer or shorter values than the previous value. In a second step, segmental interval data surrounding the decelerating and accelerating anchors were collected. In a third step, the aforementioned segments were aligned at the accelerating and decelerating anchors, and the phase-rectified signal averaging signal was obtained by averaging the signals of segments (Bauer et al., 2006a; Bauer et al., 2006b; Nasario-Junior et al., 2014). The HRV indicators, DC and AC were analysed by Kubios HRV Premium software (version 3.5, https://www.kubios.com Magi Kubios Oy, Kuopio, Finland) (Tarvainen et al., 2014).

### Statistical analysis

The estimate of sample size was based on a previously published study (Wang et al., 2021), and no specific statistical methods were used to determine sample size. Data normality was analysed using the Shapiro‒Wilk test. The continuous data are described as the mean ± standard deviation or median [1st quartile, 3rd quartile], and the counting data are described as the frequency and percentage. The optimal cutoff values of the HRV indicators, DC and AC for binary classification were obtained by X-tile software (Robert L. Camp, Yale University, New Haven, Connecticut, United States), which performs standard Monte Carlo tests to produce corrected *p* values, thus assessing the statistical significance of data evaluated by multiple cutoffs (Camp et al., 2004). The Kaplan-Meier method was used to construct survival curves and evaluate median survival. Univariate Cox regression analysis was used for clinical indicators. Considering the correlation among HRV, DC and AC, we performed a multivariate Cox regression analysis for each HRV indicator, DC and AC individually with the clinical prognostic factors that had statistical significance in univariate Cox regression analysis. The statistical analysis was performed using SPSS 25.0 software (IBM Corp., Chicago, Illinois, United States of America), and statistical significance was defined as *p* < 0.05.

## Results

In this study, electrocardiogram data from 40 patients with ES-SCLC were collected, and 1 patient was excluded due to missing data. Thirty-nine patients (mean age 62.7 ± 8.1 years) were enrolled in the final analysis. Table 1 shows the general characteristics of patients with ES-SCLC. Thirty-three patients (84.6%) died, and 6 patients (15.4%) survived. The median follow-up time was 42.2 months, and the range of follow-up time was 1.0–44.4 months.

**TABLE 1:** Basic characteristics of the enrolled ES-SCLC patients.

Characteristics	All (N = 39)
Sex	
Female	13 (33.3%)
Male	26 (66.7%)
Age (year)	62.7 ± 8.1
BMI (kg/m^2^)	23.4 ± 3.6
Mean HR (bpm)	82.9 ± 12.3
RR (Hz)	0.31 ± 0.08
Smoking	
No	27 (69.2%)
Yes	12 (30.8%)
KPS	
≤ 70	9 (23.1%)
> 70	30 (76.9%)
Radiotherapy	
Without	2 (5.1%)
With	37 (94.9%)
Chemotherapy	
Without	3 (7.7%)
With	36 (92.3%)
Targeted therapy	
Without	33 (84.6%)
With	6 (15.4%)
Immunotherapy	
Without	36 (92.3%)
With	3 (7.7%)
Surgery	
Without	39 (100%)
With	0 (0%)
SDNN (ms)	15.0 [11.2, 20.6]
RMSSD (ms)	7.9 [4.4, 13.6]
DC (ms)	7.1 [2.9, 15.0]
AC (ms)	−7.1 [-14.7, −2.9]

Values are expressed as the number of patients (percentage), mean ± standard deviation or median [1st quartile, 3rd quartile].

Abbreviations: BMI, body mass index; Mean HR, mean heart rate; bpm, beats per minute; RR, respiration rate; KPS, Karnofsky performance status; SDNN, standard deviation of the normal-normal intervals; RMSSD, root mean square of successive interval differences; DC, deceleration capacity of heart rate; AC, acceleration capacity of heart rate.

Univariate analysis showed that the overall survival of ES-SCLC patients was not significantly correlated with sex, age, body mass index, mean heart rate, respiratory rate, smoking history, radiotherapy history, chemotherapy history, targeted therapy history, or immunotherapy history. Karnofsky performance status (KPS) was significantly associated with overall survival in ES-SCLC patients (hazard ratio = 2.733, 95% confidence interval (CI): 1.211–6.164, *p* = 0.015) (Table 2).

**TABLE 2:** Univariate Cox regression analyses of clinical characteristics and overall survival in ES-SCLC patients.

	Univariate analysis	
	Hazard ratio (95% CI)	*p* value
Sex		0.406
Female	0.729 (0.346, 1.536)	
Male	Ref	
Age (year)	0.992 (0.950, 1.035)	0.696
BMI (kg/m^2^)	0.932 (0.841, 1.032)	0.175
Mean HR (bpm)	1.014 (0.980, 1.050)	0.414
RR (Hz)	0.165 (0.001, 73.753)	0.563
Smoking		0.955
No	0.979 (0.464, 2.064)	
Yes	Ref	
KPS		**0.015**
≤ 70	2.733 (1.211, 6.164)	
> 70	Ref	
Radiotherapy		0.310
Without	2.157 (0.489, 9.516)	
With	Ref	
Chemotherapy		0.359
Without	1.758 (0.527, 5.868)	
With	Ref	
Targeted therapy		0.175
Without	2.286 (0.692, 7.553)	
With	Ref	
Immunotherapy		0.132
Without	4.637 (0.630, 34.145)	
With	Ref	

Bold *p* values indicate statistical significance (*p* value < 0.05).

Abbreviations: CI, confidence interval; BMI, body mass index; Mean HR, mean heart rate; bpm, beats per minute; RR, respiration rate; KPS, Karnofsky performance status.

**FIGURE 1:**
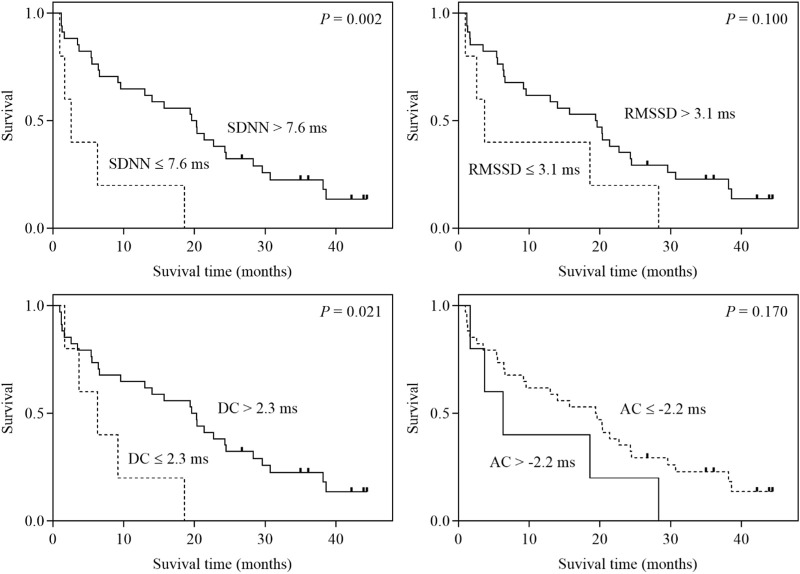
Kaplan‒Meier survival curves for ES-SCLC patients stratified by SDNN, RMSSD, DC and AC.

In the univariate analysis, the HRV parameter SDNN as well as DC were significantly correlated with the overall survival of ES-SCLC patients (all *p* < 0.05). Specifically, compared with the high-value SDNN group, the low-value SDNN group had a poorer prognosis (2.6 months vs. 19.6 months, *p* = 0.005). The ES-SCLC patients in the low DC value group had a shorter overall survival than those in the high DC value group (6.3 months vs. 19.6 months, *p* = 0.029). There was no statistically significant correlation between RMSSD or AC and the overall survival of ES-SCLC patients. In multivariate analysis, after adjusting for KPS, HRV parameters SDNN (hazard ratio = 5.254, 95% CI: 1.817–15.189, *p* = 0.002), RMSSD (hazard ratio = 3.024, 95% CI: 1.093–8.372, *p* = 0.033), as well as DC (hazard ratio = 3.909, 95% CI: 1.353–11.293, *p* = 0.012) were shown to be independent factors for predicting the overall survival of ES-SCLC patients (Table 3; Figure 1).

**TABLE 3:** Univariate and multivariate analyses of SDNN, RMSSD, DC and AC as predictors of overall survival in ES-SCLC patients.

	Median survival (M)	Univariate analysis		Multivariate analysis	
		Hazard ratio (95% CI)	*p* value	Hazard ratio (95% CI)	*p* value
SDNN (ms)			**0.005**		**0.002**
≤ 7.6	2.6	4.421 (1.576, 12.401)		5.254 (1.817, 15.189)	
> 7.6	19.6	Ref		Ref	
RMSSD (ms)			0.109		**0.033**
≤ 3.1	3.7	2.206 (0.838, 5.807)		3.024 (1.093, 8.372)	
> 3.1	19.4	Ref		Ref	
DC (ms)			**0.029**		**0.012**
≤ 2.3	6.3	3.145 (1.122, 8.810)		3.909 (1.353, 11.293)	
> 2.3	19.6	Ref		Ref	
AC (ms)			0.178		0.091
≤ −2.2	19.4	0.513 (0.195, 1.355)		0.424 (0.157, 1.146)	
> −2.2	6.3	Ref		Ref	

Bold *p* values indicate statistical significance (*p* value < 0.05).

Abbreviations: CI, confidence interval; SDNN, standard deviation of the normal-normal intervals; RMSSD, root mean square of successive interval differences; DC, deceleration capacity of heart rate; AC, acceleration capacity of heart rate.

## Discussion

The aim of this study was to explore the correlation between short-term HRV, DC and AC and overall survival in ES-SCLC patients. The results showed that the HRV parameter SDNN as well as DC were significantly correlated with the overall survival of patients with ES-SCLC. After adjusting for KPS, we found that the HRV parameters SDNN, RMSSD, as well as DC were independent prognostic factors for ES-SCLC patients.

There are complex bidirectional connections between the ANS, hypothalamic‒pituitary‒adrenal axis, and immune system, which have crucial roles in maintaining homeostasis (Mueller et al., 2022). The ANS activates the hypothalamic‒pituitary‒adrenal axis through its effector molecule norepinephrine (Valenta et al., 1986; Toufexis and Walker, 1996). It is unclear how the hypothalamic‒pituitary‒adrenal axis influences the activity of the ANS, but it may involve reduced cardiovagal baroreflex sensitivity and PNS activity (Ouyang and Wang, 2000; Adlan et al., 2018). The postganglionic neurotransmitters of the SNS and the PNS, i.e., norepinephrine and acetylcholine, can bind to specific receptors on immune cells and regulate immune reactions (Mueller et al., 2022). For example, stimulation of the vagus nerve suppresses the expression of tumour necrosis factor-alpha, interleukin-6 and additional proinflammatory cytokines (Borovikova et al., 2000; Wang et al., 2003). Vagally mediated HRV parameters were negatively associated with inflammatory markers such as interleukin-6, possibly because the cholinergic anti-inflammatory pathway may be activated by vagal stimulation, thereby inhibiting the synthesis and action of proinflammatory cytokines (Pavlov and Tracey, 2005; Williams et al., 2019; Matos et al., 2021). The activation of α- or β-adrenoceptors is related to the inflammation level (Pongratz and Straub, 2014). ANS imbalance may cause chronic inflammation, and chronic inflammation is recognized as a key factor in the development and progression of malignant tumours (Singh et al., 2019).

The SDNN reflects the overall activity of the ANS (Vanderlei et al., 2009; Lombardi and Stein, 2011). Clinical studies have found significant associations between decreased SDNN and elevated levels of inflammatory markers, cancer progression, and reduced survival in patients with malignant tumours (Hu et al., 2018; Ciurea et al., 2021; Wang et al., 2021; Wu et al., 2021). This study revealed that SDNN was independently associated with overall survival in ES-SCLC patients, suggesting that SDNN has the potential to serve as a prognostic factor in ES-SCLC patients. RMSSD reflects vagus nerve activity (Vanderlei et al., 2009; Lombardi and Stein, 2011). Gidron et al. (2018) used the ratio of RMSSD to C-reactive protein as an indicator to reflect neuroimmunomodulation. The results showed that a reduction in the value of this index predicted a shorter survival in non-small cell lung cancer patients (Gidron et al., 2018). In this study, univariate analysis showed no significant association between RMSSD and overall survival in ES-SCLC patients. However, after adjusting for KPS in multivariate analyses, we found that RMSSD was significantly associated with overall survival in ES-SCLC patients. This may be because HRV variables can be used to monitor cancer patients’ general wellbeing and ability to perform activities of daily living (Kim et al., 2015). In the univariate analysis, the true effect of RMSSD was masked by the effect of KPS; however, in the multivariate analyses, the true effect of RMSSD was revealed after adjusting for KPS. Notably, short-term SDNN values and RMSSD values were generally low in patients with ES-SCLC in this study compared to patients with other types of advanced malignancies, such as advanced breast cancer (Wang et al., 2021; Wu et al., 2021). This may be because SCLC is a neuroendocrine tumour and SCLC has an impact on electrolytes, which will greatly affect the heart rhythm (Fiordoliva et al., 2017; Wu et al., 2020).

DC and AC, emerging tools for assessing autonomic function, can be used to characterize the regulatory capacity of the PNS and SNS, respectively, offering an alternative to traditional HRV metrics (Bauer et al., 2006a; Zou et al., 2016). DC provides significant advantages over standard measures for assessing short-term HRV. DC is more robust to artefacts, noise, and nonstationarities because of its underlying signal processing algorithm. DC also captures deceleration-related oscillations of heart rate regardless of frequency, which are more closely related to vagal activity (Bauer et al., 2006a; Eick et al., 2015). Bauer et al. (2006a) found that DC was more accurate in predicting mortality after myocardial infarction than the conventional measures of HRV. Recent observational studies have shown that DC provides useful information for risk stratification in patients with malignant tumours in clinical settings (Feng and Yang, 2015; Boehm et al., 2018; Feng et al., 2021). For example, DC was an effective predictor of epirubicin-related or trastuzumab-related cardiotoxicity development in patients with breast cancer (Feng and Yang, 2015; Feng et al., 2021). Boehm et al. (2018) found that DC was an independent predictor of death during hospitalization for emergency patients with malignant diseases and that DC could independently predict the risk of death within 180 days. In our study, after adjusting for confounding factors, DC was a significant prognostic factor for patients with ES-SCLC. This suggests that reduced vagus nerve regulation (reflected by DC) in ES-SCLC patients predicts a poor prognosis. The median survival time was significantly shorter in the high AC value group than in the low AC value group (6.3 months vs. 19.4 months). However, there was no significant correlation between AC and the overall survival of ES-SCLC patients. The present study has a small sample size; thus, prospective studies with larger sample sizes are required to validate the clinical value of the current findings.

Patients with malignant tumours are prone to depression under the multiple pressures of economic burden and disease progression (Polityńska et al., 2022). Depressive mood may affect the neuro-immuno-endocrine system of patients, resulting in autonomic dysfunction, decreased immune function, and increased activation of the tumour inflammatory response (Felger et al., 2020; Ahmad et al., 2021; Polityńska et al., 2022). Chronic stress has been shown to promote invasion and distant metastasis of breast, gastric, and colon cancers by activating the SNS (Zhang et al., 2019; An et al., 2021; Guan et al., 2023). Published studies have shown that the tumour microenvironment changes under chronic stress, namely, the number and/or function of immunosuppressive cells and related cytokines increases, and the number and/or function of immunosupportive cells and related cytokines decreases (Hong et al., 2021; Tian et al., 2021). There is much recent evidence that different forms of daily biofeedback training or supportive therapy may have a positive effect on autonomic modulation or mood (Niederer et al., 2013; Lehrer and Gevirtz, 2014; Warth et al., 2015; Warth et al., 2016; Palma et al., 2020). For example, Niederer et al. (2013) have shown that exercise may enhance autonomic regulation and overall quality of life in cancer patients. Warth et al. (2015; 2016) showed that music therapy can promote relaxation and happiness in patients receiving palliative care; compared to the control group, patients who received the music intervention had a significant increase in vagally mediated HRV. HRV biofeedback training or supportive therapy to actively improve patients’ tumour-related depression and enhance ANS function are expected to be valuable supplements for cancer treatment.

Although this is a prospective study, there are some limitations. First, some background variables that might influence ANS, such as stress level and exercise volume, were not included. Second, the sample size was small, and the statistical power was limited. We did not present regression analyses with censored data, although it would be useful to see the unique variance contributed by autonomic modulation indices. To address these limitations, future studies should further clarify the correlation between short-term HRV, DC and AC and prognosis in ES-SCLC patients after expanding the sample size and controlling for more confounding factors.

## Conclusion

This study showed that the short-term HRV parameters SDNN, RMSSD, as well as DC are independent prognostic factors for ES-SCLC patients, suggesting that ANS function (indicated by HRV and DC) might be a novel predictive biomarker for prognosis in patients with ES-SCLC.

## Data Availability

The raw data supporting the conclusion of this article will be made available by the authors, without undue reservation.
